# Epidemiology and clinical features of Birt-Hogg-Dubé syndrome: A nationwide population-based study in South Korea

**DOI:** 10.1371/journal.pone.0269358

**Published:** 2022-06-06

**Authors:** Hyung Jun Park, Ye-Jee Kim, Min-Ju Kim, Ho Cheol Kim

**Affiliations:** 1 Department of Pulmonary and Critical Care Medicine, Asan Medical Center, University of Ulsan College of Medicine, Seoul, South Korea; 2 Department of Clinical Epidemiology and Biostatistics, Asan Medical Center, University of Ulsan College of Medicine, Seoul, South Korea; Seoul National University College of Medicine, REPUBLIC OF KOREA

## Abstract

**Background:**

Birt–Hogg–Dubé (BHD) syndrome is an ultrarare lung disease with unclear prevalence and incidence. Our study aimed to identify the epidemiological and clinical features of BHD syndrome by using nationwide claims data from the Korean Health Insurance Review and Assessment service.

**Methods:**

Patients with BHD syndrome who had the following criteria were included: 1) tested for folliculin gene mutation, and 2) had at least one of the conditions: other specified malformation syndromes, fibrofolliculoma, acrochordon, lung cyst, cancer, and pneumothorax based on International Classification of Disease–10 code.

**Results:**

We found 26 patients with BHD syndrome from 2017 to 2019. The prevalence of BHD syndrome was 5.67 per 10^7^ population, with no peak age. Among incidence cases, the median age of diagnosis was 51 years, with slightly more females than males (n = 15, 57.7%). Over half of the patients (n = 14, 53.8%) experienced pneumothorax, and 10 (38.5%) developed malignant neoplasm within the clinical course.

**Conclusions:**

The prevalence of BHD syndrome in Korea is extremely low. However, affected patients manifest several comorbidities, including malignant neoplasm and repetitive pneumothorax.

## Introduction

Birt–Hogg–Dubé (BHD) syndrome is a rare inherited autosomal dominant disorder [1]. It is believed to be caused by the germline mutation of folliculin (FLCN) gene [2, 3]. However, the exact pathogenesis and function of FLCN remains unknown. Although FLCN mutations are often found in affected family members [4, 5], de novo development of BHD syndrome with no prior family history can also occur [6].

Typically, BHD syndrome is characterized by skin lesion, renal cancer, cystic lung disease, and spontaneous pneumothorax [7]. However, the spectrum, onset time, and frequency of these clinical manifestations are diverse, making the diagnosis difficult [8, 9]. In addition, the genetic, epidemiologic, and clinical characteristics of BHD syndrome might differ between Asian and Western populations [10, 11]. Currently, the nationwide data about its incidence, prevalence, and accompanying comorbidities are unavailable. Thus, this study aimed to investigate the epidemiologic data and clinical characteristics of BHD syndrome by using the nationwide claims data of Korea.

## Materials and methods

### Study design and data collection

This nationwide retrospective cohort study enrolled patients diagnosed with BHD who also had an ICD-10 code and an insurance payment code. In South Korea, the National Health Insurance Service is a universal insurance system that provides both inpatient and outpatient healthcare services for nearly all citizens, which enables to the investigation of the prevalence of disease in the whole national population [12, 13]. Similar to previous code-based nationwide research, we conducted an analysis based on ICD-10 codes [14, 15]. Considering its extreme rarity, the BHD syndrome does not have its own ICD-10 code; rather, it belongs to the ICD code Q878, which includes Alport syndrome, arterial tortuosity syndrome, and other congenital malformation diseases. Moreover, the confirmation test, that is, the FLCN gene test, is not common; thus, it was included in the code C5808. S1 Table lists the tests included in C5808. Patients with BHD syndrome in Korea are under the insurance policy for incurable rare diseases, which have distinct codes. However, given that BHD syndrome is rare, it is included in the rare incurable disease code V900, which consists of 127 diseases (S2 Table). Considering this limitation, the BHD syndrome was also defined using the insurance payment code for FLCN gene (C5808) and the comorbidities according to the corresponding ICD codes.

From the Health Insurance and Review Agency (HIRA) database between January 2007 and October 2019, we extracted patients undergoing FLCN gene tests using national health insurance payment code (C5808) after 2016 and subclass of gene test (C5808446) after 2017. As the presentation can differ among patients due to different disease onsets per organ, we defined BHD patients as those tested for FLCN gene mutation (C5808446) and having at least one of: other specified malformation syndromes (Q87.8), fibrofolliculoma (D23), acrochordon (L918), lung cyst (Q330), renal cancer (C64, C65, and cancer registration code (V027, V193, and V194)), and pneumothorax (J93) based on International Classification of Disease-10 (ICD-10). For distinction from other cystic lung diseases, the following diseases were excluded: Langerhans cell histiocytosis (J848) and lymphangioleiomyomatosis (D181). In BHD patients, demographic characteristics and associated diseases including malignancy were described. To identify the diseases associated with BHD syndrome, we extracted diagnoses within 1 year before and after the FLCN gene mutation test. To protect individual privacy, anonymized and de-identified information was analyzed. To quantity pneumothorax in BHD syndrome, patient-wise pattern of hospitalization was depicted throughout the entire studied period.

### Ethics approval and consent to participate

Not required, because this study only use publicly available Health Insurance and Review Agency database.

### Statistical methods

**Fig 1:**
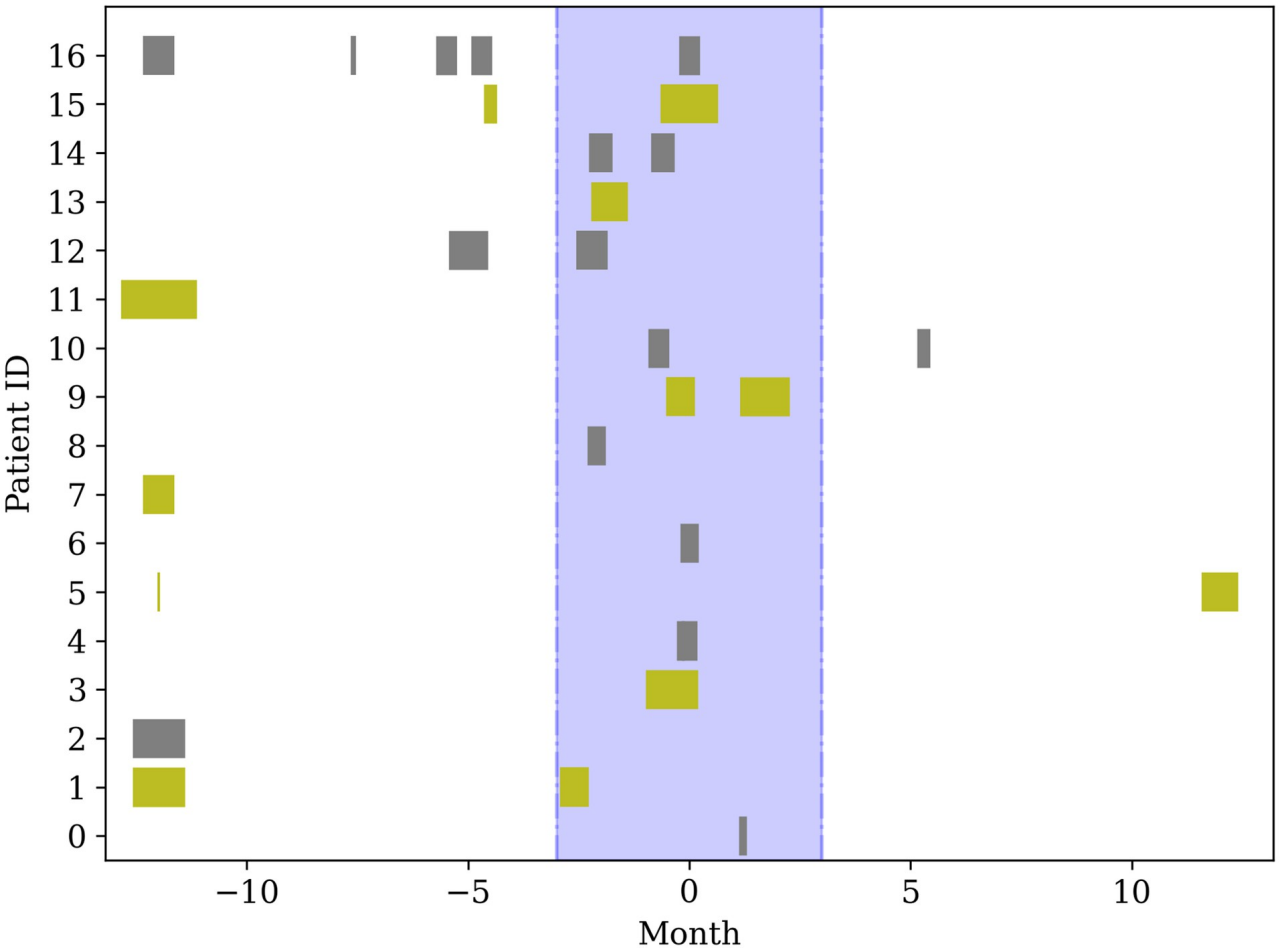
Hospitalization for pneumothorax compared with date of BHD diagnosis. The figure shows the onset and in-hospital period for pneumothorax per patient. The month zero is defined by the day of BHD diagnosis, and the shaded area is the previous 3 months of the diagnosis. The hospital admission period is depicted as the length of each bar.

The patients’ age was described using median and 25% and 75% quantiles. To understand the natural course of cancer risks, we showed the cumulative incidence using number and percentage. As the number of included patients was small, we could not evaluate group differences by usual methods [16]. The temporal relationship between the date of FLCN gene claim and pneumothorax, which is a clue for diagnosing BHD, is shown in Fig 1.

## Results

### Prevalence of BHD syndrome

Although the FLCN gene test was introduced in 2014 in South Korea, this test was first claimed in January 2016. In the database, 26 patients had BHD syndrome (5.67 per 10 million). Among them, the number and prevalence by sex were 11 and 5.67 (95% CI: 3.71–8.32) in males and 15 and 6.60 (95% CI: 3.7–10.9) in females, respectively. BHD syndrome tended to develop more in females than in males. The median (interquartile range) age was 51 (34–58) years, with 46 (30–57) years in males and 53.5 (31–59) years in females specifically. Moreover, 11 of 26 patients (42.3%) with BHD syndrome underwent FLCN gene test around 50–59 years of age and 8 of 26 (30.8%) patients were tested around 30–49 years of age. The distribution of diagnosis age according to sex is described in Table 1.

**Table 1:** The number of BHD patients according to age group and sex.

Age group	Total		Male		Female	
	N	Prevalence 95% CI	N	Prevalence 95% CI	N	Prevalence 95% CI
10–19	1	2.01 (0.05–11.2)	0	0 NA	1	4.18 (0.11–23.3)
20–29	3	4.40 (0.91–12.8)	2	5.58 (0.68–20.1)	1	3.09 (0.08–17.2)
30–39	4	5.65 (1.54–14.4)	2	5.50 (0.67–19.9)	2	5.81 (0.7–21)
40–49	4	4.77 (1.3–12.22)	2	4.69 (0.57–16.9)	2	4.85 (0.59–17.5)
50–59	11	12.69 (6.34–22.7)	4	9.16 (2.5–23.4)	7	16.27 (6.54–33.5)
60–69	2	3.16 (0.38–11.4)	1	3.24 (0.08–18.0)	1	3.10 (0.08–17.2)
70–79	1	2.78 (0.07–15.4)	0	0 NA	1	5.02 (0.13–28.0)
total	26	5.67 (3.71–8.32)	11	4.76 (2.38–8.52)	15	6.60 (3.7–10.9)

NA: Not applicable; N: Number of BHD patients; Prevalence: calculated per 10 million

### Comorbidities

Among the 26 BHD patients, 10 patients had malignancy in this cohort observational period. Among the patients, nine (34.6%) had malignancy before the FLCN gene test and one was diagnosed with cancer after this test (Table 2). In terms of cancer subtypes, digestive cancer (15.4%) and urologic cancer (11.5%) showed the first and second highest incidence before and after BHD syndrome diagnosis, respectively. Lip and orophaynx, thoracic, genital, and hematologic cancers were also identified. Table 3 summarizes the associated diseases identified within 1 year before and after the FLCN gene test. The common comorbidities other than respiratory disease according to organ were gingivitis and periodontal disease (84%) and gastritis and duodenitis (88%). We also found gastroesophageal reflux disease (65.4%), ophthalmic disease (53.8%), dyslipidemia (53.8%), allergic contact dermatitis (42.3%), and hypertension (38.5%) (Table 3). Meanwhile, four patients (16%) had fibrofolliculoma identified within 1 year of the FLCN gene test.

**Table 2:** Associated malignancy in patients with BHD syndrome before and after folliculin gene test.

ICD-10 code	Cancer type	Before (N = 26)	After (N = 26)	Total (N = 26)
C00–C97	All kinds of malignancy	9 (34.6%)	1 (3.8%)	10 (38.5%)
C15–C26	Digestive	3 (11.5%)	1 (3.8%)	4 (15.4%)
C64–C68	Ureter and bladder	3 (11.5%)	0 (0%)	3 (11.5%)
C00– C14	Lip and oropharynx	2 (7.7%)	0 (0%)	2 (7.7%)
C30–C39	Respiratory and intrathoracic	1 (3.8%)	0 (0%)	1 (3.8%)
C51–C63	Genital	1 (3.8%)	0 (0%)	1 (3.8%)
C81–C96	Hematologic	1 (3.8%)	0 (0%)	1 (3.8%)

The date of malignancy contains all periods of the patients before and after the folliculin gene mutation test.

**Table 3:** Associated diseases before and after the diagnosis of BHD syndrome.

ICD-10 code	Associated disease	Total (N = 26) (Number, %)
K29	Gastritis and duodenitis	23 (88.5%)
J00-98	Upper respiratory disease	23 (88.5%)
K02-05	Gingivitis and periodontal disease	22 (84.6%)
J20	Acute bronchitis	18 (69.2%)
K21	Gastroesophageal reflux disease	17 (65.4%)
J93	Pneumothorax	14 (53.8%)
H04, H10, H52	Ophthalmic disease	14 (53.8%)
E78	Dyslipidemia	14 (53.8%)
L03, L23, L50, M79	Skin disease	11 (42.3%)
J84	Other interstitial lung diseases	10 (38.5%)
I10	Hypertension	10 (38.5%)

The date of the associated disease contains only within 1 year before and after the folliculin gene test. Upper respiratory disease includes following codes: J00, J01, J02, J03, J04, J06, J20, J30, J40, J44, and J98.

### Pneumothorax

During the study period, 17 (65.4%) patients suffered at least one pneumothorax, 10 of whom (58.8%) had recurrent pneumothorax (Table 4). Pneumothorax was more common in females than in males (11, 64.7% vs. 6, 35.2%). The median age of patients at pneumothorax diagnosis was 43 (31–57) years (25%–75%), with 40 (28–57) years in males and 43 (34–55) years in females specifically. Pneumothorax occurring more than once was found in 5 of 6 male patients (83%) and in 5 of 11 female patients (45%). In those with at least one pneumothorax event, the mean (standard deviation) length of hospital stay was 9.9 (6.1) days in the first event and 12.6 (6.8) days in the second event. All the patients with recurrent pneumothorax (n = 10), no patients underwent surgery and 70% (n = 7/10) were treated with chest tube insertion. The median length of hospital stay by sex was 8 (6.5–13) in males and 9.5 (5.75–15.25) in females. Pneumothorax that occurred within 3 months of BHD syndrome diagnosis was found in 13 of all 26 patients (Fig 1). The median (interquartile range) time from pneumothorax to BHD syndrome diagnosis was 39 (5.4–98) months.

**Table 4:** Number of pneumothoraxes among BHD patients.

Number of pneumothoraxes	Number of patients	%
0	9	
1	7	26.9%
2	9	34.6%
>3	1	3.8%
Total	26	

## Discussion

The current study is the first population-based research performed on the prevalence of BHD syndrome in Korea by using a nationwide claims database. Although the prevalence of BHD syndrome was extremely rare in South Korea (5.67 per 10 million), various comorbidities, including malignancy and repetitive pneumothorax, were found.

Given that the BHD syndrome is a rare inherited disorder [17], its incidence and prevalence remain unknown. Several previous reports focused on the prevalence of BHD syndrome in patients with spontaneous pneumothorax [18, 19] or family history [5, 15, 16]. Recently, Hu et al. conducted a literature review of a large BHD syndrome cohort (120 families with 221 cases) in China [20]. However, they collected information using only published data; hence, the research was not representative of the nationwide data. In South Korea, Lee et al. reported only 12 patients (10 patients confirmed by FLCN gene test) who had BHD syndrome in a single largest tertiary hospital [21], suggesting the rarity of this condition. Our current data showed that BHD syndrome is ultrarare in South Korea, with a prevalence rate of only 5.67 per 10 million. As mentioned earlier, defining BHD syndrome cases using the ICD code alone seemed inadequate because it might omit other patients with BHD syndrome, leading to bias in evaluating epidemiologic data. In addition, patients with BHD syndrome who did not undergo the FLCN gene test were excluded in our analysis; this exclusion might have led to the underestimation of actual epidemiology. However, our data might serve as a reference for understanding this rare disease in South Korea.

Clinical manifestations of patients with BHD syndrome may also differ in terms of ethnicity [20, 22]. Kunogi et al. evaluated 30 Japanese patients with BHD syndrome and reported that while nearly all patients (96.7%) experienced pneumothorax, only 7 (23.3%) had skin lesion, and 2 (6.7%) had renal tumor [22]. Hu et al. investigated 221 Chinese patients with BHD syndrome and showed prevalence of pneumothorax (71.0%), skin lesion (18.1%), and renal cancer (3.6%) [20]. Thus, Asian patients might have a higher prevalence of pneumothorax and a lower incidence of cutaneous and renal manifestations than Western patients [4, 23–25]. Similarly, our current study found that pneumothorax occurred in over half of the patients (58.8%), but skin lesion (fibrofolliculoma, trichodiscoma, or accrocardon) and ureter and bladder cancer only accounted for 9 (34.6%) and 3 patients (11.5%), respectively. Recently, Liu et al. analyzed 51 Chinese patients with BHD syndrome and showed that Chinese patients had FLCN gene mutant loci that were different from those of Western patients [11]. This observation might possibly explain the ethnic difference. Notably, our current study showed several comorbidities, especially malignant neoplasm, in patients with BHD syndrome. Various tumors other than renal cancer might also occur in these patients [5, 24, 26, 27]. However, the gold standard or guidelines on how to optimally screening and manage these patients are still unavailable. Hence, further studies are needed.

Patients with BHD syndrome are predisposed to pneumothorax [1, 7]. Zbar et al. reported that the odds ratio for spontaneous pneumothorax in patients with BHD syndrome was 32 when compared with that in the general population [28]. In the current study, 65.4% of the patients experienced pneumothorax, comparable to other previous studies (approximately 24%–76%) [4, 10, 23, 29]. In addition, the median age of pneumothorax development was 43 years, which is also comparable to other previous studies with a relatively large number of patients [17, 23, 25]. Repetitive pneumothorax might also occur [23, 29]. Toro et al. showed that 101 episodes of pneumothorax (1 to 5 times in each patient) occurred in 48 patients with BHD syndrome [29]. In addition, Gupta et al. reported that the average number of pneumothorax was 3.6 in patients with BHD syndrome [23]. Our study population showed a similar recurrence rate of pneumothorax (58.8%). Notably, the hospital stay was longer in the second episode than in the first episode, suggesting that treatment might be difficult in patients with repetitive pneumothorax. Moreover, pneumothorax within 3 months of BHD syndrome diagnosis occurred in 13 patients (50% of study population), consistent with the previous study of Gupta et al. [23], which showed that pneumothorax was the presenting manifestation of BHD in 65% of their patients.

This study has some limitations. Considering that it used a nationwide medical claims database, BHD syndrome without FLCN gene mutation is not included in our dataset, thereby possibly omitting approximately 10% of patients with BHD syndrome [30]. Our study also did not include those with a family history of BHD syndrome and FLCN gene mutation, thereby reducing the representation of genetic features. Furthermore, if the clinician did not perform FLCN gene test for patients with suspected BHD syndrome, our criteria would underestimate the real prevalence. However, this study collected all FLCN gene tests performed in South Korea from 2016 to 2019, but only 26 patients were found. Moreover, we collected a maximum of 12.8-year follow-up data of patients suspected with BHD syndrome, including their clinical phenotypes combined with comorbidities and pneumothorax history, thereby showing the characteristics and clinical course of BHD syndrome.

## Conclusions

In conclusion, the prevalence of BHD syndrome in South Korea is low. The patients with BHD syndrome are characterized by several comorbidities, including malignant neoplasm and repetitive pneumothorax.

## Supporting information

S1 Table Tests included in C5808 codes. (DOCX)

S2 Table Diseases included in rare incurable disease code. NA: not applicable due to absence of a defined code.
(DOCX)

## References

[1] Daccord C, Good JM, Morren MA, Bonny O, Hohl D, Lazor R. Birt–hogg–dubé syndrome. Eur Respir Rev. 2020;29: 1–14. doi: 10.1183/16000617.0042-2020 PMC9489184 32943413

[2] Khoo SK, Bradley M, Wong FK, Hedblad MA, Nordenskjöld M, Teh BT. Birt-Hogg-Dubé syndrome: mapping of a novel hereditary neoplasia gene to chromosome 17p12-q11.2. Oncogene. 2001;20: 5239–5242. doi: 10.1038/sj.onc.1204703 11526515

[3] Schmidt LS, Warren MB, Nickerson ML, Weirich G, Matrosova V, Toro JR, et al. Birt-Hogg-Dubé syndrome, a genodermatosis associated with spontaneous pneumothorax and kidney neoplasia, maps to chromosome 17p11.2. Am J Hum Genet. 2001;69: 876–882. doi: 10.1086/323744 11533913 PMC1226073

[4] Schmidt LS, Nickerson ML, Warren MB, Glenn GM, Toro JR, Merino MJ, et al. Germline BHD-mutation spectrum and phenotype analysis of a large cohort of families with Birt-Hogg-Dubé syndrome. Am J Hum Genet. 2005;76: 1023–1033. doi: 10.1086/430842 15852235 PMC1196440

[5] Leter EM, Koopmans AK, Gille JJP, van Os TAM, Vittoz GG, David EFL, et al. Birt-Hogg-Dubé syndrome: clinical and genetic studies of 20 families. J Invest Dermatol. 2008;128: 45–49. doi: 10.1038/sj.jid.5700959 17611575

[6] Menko FH, Johannesma PC, van Moorselaar RJA, Reinhard R, van Waesberghe JH, Thunnissen E, et al. A de novo FLCN mutation in a patient with spontaneous pneumothorax and renal cancer; A clinical and molecular evaluation. Fam Cancer. 2013;12: 373–379. doi: 10.1007/s10689-012-9593-8 23264078

[7] Menko FH, van Steensel MAM, Giraud S, Friis-Hansen L, Richard S, Ungari S, et al. Birt-Hogg-Dubé syndrome: diagnosis and management. Lancet Oncol. 2009;10: 1199–1206. doi: 10.1016/S1470-2045(09)70188-3 19959076

[8] Janitzky A, Reiher F, Porsch M, Grube C, Evert M, Liehr U-B. An unusual case of Birt-Hogg-Dube syndrome with renal involvement. Urol J. 2008;5: 272–274. 19101904

[9] Kluijt I, de Jong D, Teertstra HJ, Axwijk PH, Gille JJP, Bell K, et al. Early onset of renal cancer in a family with Birt-Hogg-Dubé syndrome. Clin Genet. 2009;75: 537–543. doi: 10.1111/j.1399-0004.2009.01159.x 19320655

[10] Furuya M, Yao M, Tanaka R, Nagashima Y, Kuroda N, Hasumi H, et al. Genetic, epidemiologic and clinicopathologic studies of Japanese Asian patients with Birt–Hogg–Dubé syndrome. Clin Genet. 2016;90: 403–412. doi: 10.1111/cge.12807 27220747

[11] Liu Y, Xu Z, Feng R, Zhan Y, Wang J, Li G, et al. Clinical and genetic characteristics of chinese patients with Birt-Hogg-Dubé syndrome. Orphanet J Rare Dis. 2017;12: 104. doi: 10.1186/s13023-017-0656-7 28558743 PMC5450333

[12] Woo A, Lee SW, Koh HY, Kim MA, Han MY, Yon DK. Incidence of cancer after asthma development: 2 independent population-based cohort studies. J Allergy Clin Immunol. 2021;147: 135–143. doi: 10.1016/j.jaci.2020.04.041 32417133

[13] Yoon HJ, Kim JH, Seo GH, Park H. Risk of cancer following the use of N-nitrosodimethylamine (NDMA) contaminated ranitidine products: A nationwide cohort study in South Korea. J Clin Med. 2021;10: 1–8. doi: 10.3390/jcm10010153 33466237 PMC7795144

[14] Kim SY. Nationwide COVID-19 vaccination coverage and COVID-19 incidence in South Korea, January 2022: a national official report. 2022;2: 1–9.

[15] Yoo IK, Marshall DC, Cho JY, Yoo HW, Lee SW. N-Nitrosodimethylamine-contaminated ranitidine and risk of cancer in South Korea: a nationwide cohort study. Life Cycle. 2021;1: 1–17. doi: 10.54724/lc.2021.e1

[16] Lee SW. Methods for testing statistical differences between groups in medical research: statistical standard and guideline of Life Cycle Committee. 2022; 1–8.

[17] Toro JR, Glenn G, Duray P, Darling T, Weirich G, Zbar B, et al. Birt-Hogg-Dubé syndrome: a novel marker of kidney neoplasia. Arch Dermatol. 1999;135: 1195–1202. doi: 10.1001/archderm.135.10.1195 10522666

[18] Muller ME, Daccord C, Taffé P, Lazor R. Prevalence of Birt-Hogg-Dubé Syndrome Determined Through Epidemiological Data on Spontaneous Pneumothorax and Bayes Theorem. Front Med. 2021;8: 1–12. doi: 10.3389/fmed.2021.631168 33987191 PMC8111214

[19] Johannesma PC, Reinhard R, Kon Y, Sriram JD, Smit HJ, Van Moorselaar RJA, et al. Prevalence of Birt-Hogg-Dubé syndrome in patients with apparently primary spontaneous pneumothorax. Eur Respir J. 2015;45: 1191–1194. doi: 10.1183/09031936.00196914 25537564

[20] Hu X, Zhang G, Chen X, Xu KF. Birt–Hogg–Dubé syndrome in Chinese patients: a literature review of 120 families. Orphanet J Rare Dis. 2021;16: 1–8. doi: 10.1186/s13023-021-01848-8 34001170 PMC8130425

[21] Skolnik K, Tsai WH, Dornan K, Perrier R, Burrowes PW, Davidson WJ. Birt-Hogg-Dubé syndrome: A large single family cohort. Respir Res. 2016;17: 1–7. doi: 10.1186/s12931-016-0339-2 26928018 PMC4770529

[22] Kunogi M, Kurihara M, Ikegami TS, Kobayashi T, Shindo N, Kumasaka T, et al. Clinical and genetic spectrum of Birt-Hogg-Dubé syndrome patients in whom pneumothorax and/or multiple lung cysts are the presenting feature. J Med Genet. 2010;47: 281–287. doi: 10.1136/jmg.2009.070565 20413710 PMC2981024

[23] Gupta N, Kopras EJ, Henske EP, James LE, El-Chemaly S, Veeraraghavan S, et al. Spontaneous pneumothoraces in patients with Birt-Hogg-Dubé syndrome. Ann Am Thorac Soc. 2017;14: 706–713. doi: 10.1513/AnnalsATS.201611-886OC 28248571 PMC5427741

[24] Toro JR, Wei MH, Glenn GM, Weinreich M, Toure O, Vocke C, et al. BHD mutations, clinical and molecular genetic investigations of Birt-Hogg-Dubé syndrome: A new series of 50 families and a review of published reports. J Med Genet. 2008;45: 321–331. doi: 10.1136/jmg.2007.054304 18234728 PMC2564862

[25] Houweling AC, Gijezen LM, Jonker MA, Van Doorn MBA, Oldenburg RA, Van Spaendonck-Zwarts KY, et al. Renal cancer and pneumothorax risk in Birt-Hogg-Dubé syndrome; An analysis of 115 FLCN mutation carriers from 35 BHD families. Br J Cancer. 2011;105: 1912–1919. doi: 10.1038/bjc.2011.463 22146830 PMC3251884

[26] Vincent A, Farley M, Chan E, James WD. Birt-Hogg-Dubé syndrome: two patients with neural tissue tumors. J Am Acad Dermatol. 2003;49: 717–719. doi: 10.1067/s0190-9622(03)01583-4 14512924

[27] Palmirotta R, Donati P, Savonarola A, Cota C, Ferroni P, Guadagni F. Birt-Hogg-Dubé (BHD) syndrome: Report of two novel germline mutations in the folliculin (FLCN) gene. Eur J Dermatology. 2008;18: 382–386. doi: 10.1684/ejd.2008.0431 18573707

[28] Zbar B, Alvord WG, Glenn G, Turner M, Pavlovich CP, Schmidt L, et al. Risk of renal and colonic neoplasms and spontaneous pneumothorax in the Birt-Hogg-Dubé syndrome. Cancer Epidemiol biomarkers Prev a Publ Am Assoc Cancer Res cosponsored by Am Soc Prev Oncol. 2002;11: 393–400. 11927500

[29] Toro JR, Pautler SE, Stewart L, Glenn GM, Weinreich M, Toure O, et al. Lung cysts, spontaneous pneumothorax, and genetic associations in 89 families with birt-Hogg-Dubé syndrome. Am J Respir Crit Care Med. 2007;175: 1044–1053. doi: 10.1164/rccm.200610-1483OC 17322109 PMC1899269

[30] Radzikowska E, Lechowicz U, Winek J, Opoka L. Novel folliculin gene mutations in Polish patients with Birt–Hogg–Dubé syndrome. Orphanet J Rare Dis. 2021;16: 1–10. doi: 10.1186/s13023-021-01931-0 34229741 PMC8258955

