# Supplementary material for: Epidemiology and clinical features of Birt-Hogg-Dubé syndrome: A nationwide population-based study in South Korea
Source: PLoS One. 2022 Jun 6;17(6):e0269358. doi: 10.1371/journal.pone.0269358 (PMC9170097; doi:10.1371/journal.pone.0269358)
Supplement: S1 Table — (DOCX) [file pone.0269358.s001.docx]

**S1 Table. Tests included in C5808 codes**

| Codes | Codes Name |
| --- | --- |
| C5808356 | Genetic Tests for Germline Variants_Sequencing[ACADM Gene] |
| C5808366 | Genetic Tests for Germline Variants_Sequencing[ALB Gene] |
| C5808376 | Genetic Tests for Germline Variants_Sequencing[AR Gene] |
| C5808386 | Genetic Tests for Germline Variants_Sequencing[BTK Gene] |
| C5808396 | Genetic Tests for Germline Variants_Sequencing[COMP Gene] |
| C5808406 | Genetic Tests for Germline Variants_Sequencing[EXT1 Gene] |
| C5808416 | Genetic Tests for Germline Variants_Sequencing[EXT2 Gene] |
| C5808426 | Genetic Tests for Germline Variants_Sequencing[F11 Gene] |
| C5808436 | Genetic Tests for Germline Variants_Sequencing[FGFR3 Gene] |
| **C5808446** | **Genetic Tests for Germline Variants_Sequencing[FLCN Gene]** |
| C5808456 | Genetic Tests for Germline Variants_Sequencing[FOXP3 Gene] |
| C5808466 | Genetic Tests for Germline Variants_Sequencing[GBE1 Gene] |
| C5808476 | Genetic Tests for Germline Variants_Sequencing[GCDH Gene] |
| C5808486 | Genetic Tests for Germline Variants_Sequencing[GNAS Gene] |
| C5808496 | Genetic Tests for Germline Variants_Sequencing[GNE Gene] |
| C5808506 | Genetic Tests for Germline Variants_Sequencing[HADHB Gene] |
| C5808516 | Genetic Tests for Germline Variants_Sequencing[KCNH2 Gene] |
| C5808526 | Genetic Tests for Germline Variants_Sequencing[KCNQ1 Gene] |
| C5808536 | Genetic Tests for Germline Variants_Sequencing[LDLR Gene] |
| C5808546 | Genetic Tests for Germline Variants_Sequencing[MAPT Gene] |
| C5808556 | Genetic Tests for Germline Variants_Sequencing[MCCC2 Gene] |
| C5808566 | Genetic Tests for Germline Variants_Sequencing[MPL Gene] |
| C5808576 | Genetic Tests for Germline Variants_Sequencing[MSH6 Gene] |
| C5808586 | Genetic Tests for Germline Variants_Sequencing[MTM1 Gene] |
| C5808596 | Genetic Tests for Germline Variants_Sequencing[MUT Gene] |
| C5808606 | Genetic Tests for Germline Variants_Sequencing[NAGLU Gene] |
| C5808616 | Genetic Tests for Germline Variants_Sequencing[NF2 Gene] |
| C5808626 | Genetic Tests for Germline Variants_Sequencing[NOTCH3 Gene] |
| C5808636 | Genetic Tests for Germline Variants_Sequencing[NTRK1 Gene] |
| C5808646 | Genetic Tests for Germline Variants_Sequencing[PKD2 Gene] |
| C5808656 | Genetic Tests for Germline Variants_Sequencing[PROS1 Gene] |
| C5808666 | Genetic Tests for Germline Variants_Sequencing[RAF1 Gene] |
| C5808676 | Genetic Tests for Germline Variants_Sequencing[SGCE Gene] |
| C5808686 | Genetic Tests for Germline Variants_Sequencing[SLC26A4 Gene] |
| C5808696 | Genetic Tests for Germline Variants_Sequencing[SMAD4 Gene] |
| C5808706 | Genetic Tests for Germline Variants_Sequencing[TGFBR2 Gene] |
| C5808716 | Genetic Tests for Germline Variants_Sequencing[WAS Gene] |
| C5808726 | Genetic Tests for Germline Variants_Sequencing[SLC3A1 Gene] |
| C5808736 | Genetic Tests for Germline Variants_Sequencing[FANCG Gene] |
| C5808746 | Genetic Tests for Germline Variants_Sequencing[ZEB2 Gene] |
| C5808756 | Genetic Tests for Germline Variants_Sequencing[CBS Gene] |
| C5808766 | Genetic Tests for Germline Variants_Sequencing[MUTYH Gene] |
| C5808776 | Genetic Tests for Germline Variants_Sequencing[G6PD Gene] |
