# Supplementary material for: Epidemiology and clinical features of Birt-Hogg-Dubé syndrome: A nationwide population-based study in South Korea
Source: PLoS One. 2022 Jun 6;17(6):e0269358. doi: 10.1371/journal.pone.0269358 (PMC9170097; doi:10.1371/journal.pone.0269358)
Supplement: S2 Table — NA: not applicable due to absence of a defined code. (DOCX) [file pone.0269358.s002.docx]

**S2 Table. Diseases included in rare incurable disease code.**

| **Insurance code** | **Disease name** | **Rare incurable disease code** |
| --- | --- | --- |
| A31.9 | Atypical mycobacteriosis, familial, x-linked | V900 |
| D68.5 | Antithrombin deficiency | V900 |
| D68.5 | Protein C deficiency | V900 |
| D68.5 | Protein S deficiency | V900 |
| E16.10 | Congenital hyperinsulinaemia | V900 |
| E20.1 | Pseudohypoparathyroidism | V900 |
| E83.2 | Acrodermatitis enteropathica | V900 |
| E88.0 | α-1-Antitrypsin deficiency | V900 |
| E88.1 | Congenital systemic lipodystrophy | V900 |
| G25.8 | Stiff-man syndrome | V900 |
| H35.05 | Eales’ disease | V900 |
| H51.8 | Ocular motor apraxia, Cogan type | V900 |
| I47.2 | Catecholaminergic polymorphic ventricular tachycardia | V900 |
| K00.51 | Dentinogenesis imperfecta | V900 |
| P35.0 | Congenital rubella syndrome | V900 |
| Q04.3 | Lissencephaly | V900 |
| Q04.3 | Cerebellar agenesis | V900 |
| Q11.2 | Lenz microphthalmia syndrome | V900 |
| Q14.1 | X-linked juvenile retinoschisis | V900 |
| Q28.2 | Wyburn Mason syndrome | V900 |
| Q44.7 | Alagille’s syndrome | V900 |
| Q61.9 | Meckel syndrome | V900 |
| Q74.0 | Cleidocranial dysostosis | V900 |
| Q78.5 | Metaphyseal chondrodysplasia, Schmid type | V900 |
| Q78.9 | Pseudoachondroplastic dysplasia | V900 |
| Q80.2 | Lamellar ichthyosis | V900 |
| Q80.3 | Congenital bullous ichthyosiform erythroderma | V900 |
| Q81.0 | Epidermolysis bullosa simplex | V900 |
| Q82.3 | Incontinentia Pigmenti | V900 |
| Q82.4 | Ectodermal dysplasia (anhidrotic) | V900 |
| Q87.3 | Beckwith-Wiedemann syndrome | V900 |
| Q92.2 | Trisomy 10p | V900 |
| Q93.3 | Wolff-Hirschhorn syndrome | V900 |
| Q93.5 | 18q monosomy | V900 |
| NA | Alstrom syndrome | V900 |
| NA | Arthrogryposis, renal tubular dysfunction, and cholestasis (ARC syndrome) | V900 |
| NA | Cowden syndrome | V900 |
| NA | Dent disease | V900 |
| NA | Glucose transporter type1 (GLUT1) deficiency | V900 |
| NA | KID syndrome (Keratitis-ichthyosis-deafness) | V900 |
| NA | Kabuki syndrome | V900 |
| NA | Gorham-Stout disease (GSD) | V900 |
| NA | Campomelic dysplasia | V900 |
| NA | Multiple epiphyseal dysplasia (MED) | V900 |
| NA | Denys-Drash syndrome | V900 |
| NA | Craniometaphyseal dysplasia | V900 |
| NA | Rasmussen's encephalitis | V900 |
| NA | Langer-Giedion syndrome | V900 |
| NA | Miller-Dieker syndrome | V900 |
| NA | Congenital amegakaryocytic thrombocytopenia | V900 |
| NA | Alexander disease | V900 |
| NA | Antley-Bixler syndrome | V900 |
| NA | Congenital Ichthyosis | V900 |
| NA | Erdheim-Chester disease | V900 |
| NA | Intestinal lymphangiectasia | V900 |
| NA | Joubert syndrome | V900 |
| NA | Gitelman syndrome | V900 |
| NA | Canavan disease | V900 |
| NA | Cadasil | V900 |
| NA | Currarino syndrome | V900 |
| NA | Cronkhite-Canada syndrome | V900 |
| NA | Tufting enteropathy (Intestinal epithelial dyspalsia) | V900 |
| NA | Parry-Romberg syndrome (Progressive hemifacial atrophy) | V900 |
| NA | Fraser syndrome | V900 |
| NA | Hay-Wells syndrome | V900 |
| NA | Allan-Herndon-Dudley syndrome | V900 |
| NA | Pallister-Killian syndrome | V900 |
| NA | Cohen syndrome | V900 |
| NA | Progressive familial intrahepatic cholestasis | V900 |
| NA | Schwachman-Diamond syndrome | V900 |
| NA | Adult-onset leukoencephalopathy with axonal spheroids and pigmented glia (ALSP) | V900 |
| NA | Familial hypercholesterolemia homozygote | V900 |
| NA | Iron-refractory iron deficiency anemia | V900 |
| NA | Haddad syndrome | V900 |
| NA | Pearson syndrome | V900 |
| NA | 3MC syndrome (Malpuech-Michels-Mingarelli-Carnevale) | V900 |
| NA | Walker-Warburg syndrome | V900 |
| NA | Coffin Siris syndrome | V900 |
| NA | Alternating hemiplegia of childhood | V900 |
| NA | Schinzel Giedion syndrome | V900 |
| NA | Mowat-Wilson syndrome | V900 |
| NA | Congenital central hypoventilation syndrome | V900 |
| NA | 1p36 microdeletion syndrome | V900 |
| NA | Childhood ataxia with central nervous system hypomyelination | V900 |
| NA | Hypermanganesemia with dystonia | V900 |
| NA | 2q11 microduplication syndrome | V900 |
| NA | Distal 10q trisomy syndrome | V900 |
| NA | 15q11.2 microdeletion syndrome | V900 |
| NA | Goldberg Shprintzen syndrome | V900 |
| NA | Aicardi-Goutieres syndrome | V900 |
| NA | CARASIL syndrome (Cerebral autosomal recessive arteriopathy with subcortical infarcts and leukoencephalopathy) | V900 |
| NA | Congenital short bowel syndrome | V900 |
| NA | COL4A1-related disorder | V900 |
| NA | DYRK1A syndrome or DYRK1A-related intellectual disability syndrome | V900 |
| NA | KBG syndrome | V900 |
| NA | Kleefstra syndrome | V900 |
| NA | Pelizaeus Merzbacher disease | V900 |
| NA | Short stature with optic atrophy and Pelger-Huët anomaly (SOPH) syndrome | V900 |
| NA | Candidiasis, familial, 2, autosomal recessive (CANDF2) | V900 |
| NA | Gorlin syndrome | V900 |
| NA | Goltzs syndrome | V900 |
| NA | Rabson-Mendellhal syndrome, leprechaunism | V900 |
| NA | Leber`s hereditary optic neuropathy | V900 |
| NA | Loeys-Dietz syndrome | V900 |
| NA | ROHHAD syndrorme | V900 |
| NA | Meier-Gorlin syndrome | V900 |
| NA | Aniridia cerebellar ataxia mental deficiency | V900 |
| NA | Birt-Hogg-Dubé syndrome | V900 |
| NA | Wolfram syndrome | V900 |
| NA | Inclusion body myositis | V900 |
| NA | IPEx syndrome | V900 |
| NA | Geleophysic dysplasia | V900 |
| NA | Coffin-Lowry syndrome | V900 |
| NA | Cryopyrin associated periodic fever syndrome (CAPS) | V900 |
| NA | Idiopathic pulmonary hemosiderosis | V900 |
| NA | Diffuse pulmonary lymphangiomatosis | V900 |
| NA | Potocki-Lupski syndrome | V900 |
| NA | Floating-Harbor syndrome | V900 |
| NA | Pitt-Hopkins syndrome | V900 |
| NA | Hadju-Cheney syndrome | V900 |
| NA | Vici syndrome | V900 |
| NA | Hereditary pancreatitis | V900 |
| NA | congenital hepatic fibrosis | V900 |
| NA | Congenital chloride diarrhea (Chronic chloride diarrhea) | V900 |
| NA | Congenital Zika syndrome | V900 |
| NA | Paroxysmal Kinesigenic Dyskinesia (PKD) | V900 |
| NA | Episodic ataxia type 2 | V900 |
| NA | Orbital lymphangioma | V900 |
| NA | Chronic progressive external ophthalmoplegia | V900 |
| NA | 11p13 deletion (WAGR syndrome) | V900 |
| NA | 11q deletion syndrome (Jacobsen syndrome) | V900 |
| NA | 8q13 microdeletion syndrome (Mesomelia-synostoses syndrome) | V900 |
| NA | Deletion 8q24.1 Langer–Giedion syndrome (LGS); Trichorhinophalangeal syndrome type II (TRPS2) | V900 |
| NA | Emanuel syndrome | V900 |

NA: not applicable due to absence of a defined code
