# Supplementary material for: Epidemiology and clinical features of Birt-Hogg-Dubé syndrome: A nationwide population-based study in South Korea
Source: PLoS One. 2022 Jun 6;17(6):e0269358. doi: 10.1371/journal.pone.0269358 (PMC9170097; doi:10.1371/journal.pone.0269358)
Supplement: Attachment — Submitted filename: Response to Reviewers 0328.docx [file pone.0269358.s003.docx]

March 29^th^, 2022

Dong Keon Yon, MD, FACAAI

Academic Editor

PLOS ONE

Manuscript number: PONE-D-22-00655

Manuscript title: Epidemiology and Clinical Features of Birt-Hogg-Dubé Syndrome: A Nationwide Population-Based Study in South Korea

Dear Dong Keon Yon

We would like to thank you for the letter dated 24/02/2022, and the opportunity to resubmit a revised copy of this manuscript. We would also like to take this opportunity to express our thanks to the reviewers for the positive feedback and helpful comments for correction or modification. We have made every attempt to fully address these comments in the revised manuscript. Reviewer’s original comments are listed below, followed by our response to each comment. Changes made in the manuscript are marked in red. We very much hope the revised manuscript is accepted for publication in PLOS ONE.

The authors have declared that no competing interests exist. And the authors received no specific funding for this work. For data availability, HIRA (Health Insurance Review and Assessment Service) only provides summary data for researchers with limited permission to use the data. Therefore, we cannot share the minimal data set for this study. And we added the references which are well-performed studies using HIRA dataset which enable the national prevalence trend investigations.

Sincerely,

Ho Cheol Kim, MD, PhD

Department of Pulmonary and Critical Care Medicine,

Asan Medical Center,

88, Olympic-ro 43-gil, Songpa-gu, Seoul, 05505, South Korea

Phone No: +82 02-3010-1494

Fax No: +82 02-3010-3130

Email Address: kimhocheol10@gmail.com

Response to Reviewers

Additional Editor Comments:

#1. Please discuss and cite the papers below, in Page 3; Line 18-19.

A. Woo A, Lee SW, Koh HY, Kim MA, Han MY, Yon DK. Incidence of cancer after asthma development: 2 independent population-based cohort studies. J Allergy Clin Immunol. 2021 Jan;147(1):135-143. doi: 10.1016/j.jaci.2020.04.041. Epub 2020 May 15. PMID: 32417133.

B. Yoo IK, Marshall DC, Cho JY, Yoo HW, Lee SW. N-Nitrosodimethylamine-contaminated ranitidine and risk of cancer in South Korea: a nationwide cohort study. Life Cycle 2021;1:e1. <https://doi.org/10.54724/lc.2021.e1>

- Answer: We have added these two papers as references in page 3, line 18-21.

In South Korea, the National Health Insurance Service is a universal insurance system that provides both inpatient and outpatient healthcare services for nearly all citizens, which enables to the investigation of the prevalence of disease in the whole national population.[12,13]

Reviewer #1: The authors aimed to investigate the epidemiologic data and clinical characteristics of Birt-Hogg-Dube syndrome of Korea. The most important limitation of the study is that, apart from the 26 cases detected in the study, the number of BHD syndrome cases in which the FLCN mutation is not studied cannot be determined, and this was stated by the authors in the limitations section. Except that the article is well designed and written.

- Answer: Thank you for your comment. This study investigates the prevalence of BHD syndrome and the associating malignancy and diseases. As the BHD syndrome could be diagnosed without the FLCN gene test, we agree that your point for omitting the BHD syndrome patients without FLCN gene as we discussed in limitation section. However, we thought that this method could reflect the malignancy and associating diseases of BHD syndrome even though the limitations, which help clinicians to consider the natural history of this syndrome.

Reviewer #2: Wonderful study especially because of it’s rarity. Just out of my own interest, would like to ask a couple of questions.
1. What was the X ray and HRCT pattern of these patients.

- Unfortunately, since we used the claim data called HIRA(Health Insurance Review & Assessment) data in our current study (ref: J Lipid Atheroscler. 2022;11:e1. Understanding and Utilizing Claim Data from the Korean National Health Insurance Service (NHIS) and Health Insurance Review & Assessment (HIRA) Database for Research, by Kyoung et al.) we could not get information about the test results.

2. The study said around 58% of the patients had recurrent pneumothorax. How were they managed.

- Thanks for your comments. When we re-evaluated the patient with recurrent pneumothorax (n = 10), no patients underwent surgery and 70% (n = 7/10) were treated with chest tube insertion. We added this in the result section.
